# Protocol for the MyPREPED trial: hybrid type 2 effectiveness–implementation trial of a peer-delivered digital self-management tool (MyBRANCHES) for young people in Australia transitioning from early intervention in psychosis services

**DOI:** 10.1192/bjo.2026.12056

**Published:** 2026-07-27

**Authors:** Alyssa Milton, Ellie Brown, Urska Arnautovska, Priya Vaughan, Caroline Gao, Mary Lou Chatterton, Elizabeth Stratton, Nicola Hancock, Justin Chapman, Ravi Iyer, Simran Singh, Saahil Malhotra, Sam Batara, Symphony Chakma, Deanna De Cicco, Hadley Lindsay, Anthony Harris, Dan Siskind, Nicola Warren, Cate McHugh, Peter McArdle, Darren Phung, Jim Cook, Andrew Thompson, Nick Glozier

**Affiliations:** Faculty of Medicine and Health, University of Sydney, Australia; Australian Research Council, Centre of Excellence for Children and Families Over the Life Course, Sydney, Australia; Centre for Youth Mental Health, Faculty of Medicine, Dentistry and Health Sciences, The University of Melbourne, Australia; Faculty of Medicine, The University of Queensland, Australia; Centre for Youth Mental Health, The University of Melbourne, Australia; Orygen, The National Centre of Excellence in Youth Mental Health, University of Melbourne, Australia; Department of Epidemiology and Preventive Medicine, Monash University, Australia; School of Public Health and Preventive Medicine, Monash University, Australia; School of Health Sciences, Faculty of Medicine and Health, The University of Sydney, Australia; Metro South Addiction and Mental Health Services, Metro South Health Service District, Woolloongabba, Australia; Centre for Mental Health, School of Pharmacy and Medical Sciences, Griffith University, Australia; Swinburne University of Technology, Australia; Functional Recovery Team, Uniting NSW ACT, Sydney, Australia; Brain Dynamics Centre, Westmead Institute for Medical Research and The University of Sydney, Australia; School of Medicine, University of Queensland, Australia; Queensland Centre for Mental Health Research, Australia; School of Clinical Medicine, University of New South Wales, Australia; TechLab, University of Sydney, Australia

**Keywords:** Early psychosis, peer support, self-management, digital mental health, discharge

## Abstract

**Background:**

Peer-supported self-management at discharge from early intervention in psychosis services (EIPS) has received limited attention. The MyPREPED (My Personal Recovery Plan for Early Discharge) trial will evaluate a co-designed, digital and paper-based, peer-delivered recovery and self-management focused intervention, tailored for young people exiting EIPS.

**Aims:**

This protocol describes a hybrid type 2 effectiveness–implementation trial designed to assess MyPREPED’s impact, feasibility, real-world implementation and cost–utility.

**Method:**

This multi-site, mixed-method, two-arm (1:1), parallel-group, randomised controlled trial (MyPREPED versus treatment as usual) trial will be delivered across eight Australian EIPS that deliver ultra-high risk and/or first-episode psychosis streams, using a hybrid type 2 implementation–effectiveness design. Eligible participants are young people aged 16 years and over within 6 months of planned discharge from EIPS. Peer coaches will deliver up to ten sessions of using a self-management plan (modules: discharge, recovery, well-being, relapse prevention, goal-setting, service navigation). Co-primary outcomes include (a) mental health recovery (Recovery Assessment Scale – Domains and Stages; effectiveness outcome) and (b) feasibility (Feasibility of Implementation Measure; implementation outcome). Secondary outcomes assess broader effectiveness domains (mental health quality of life, clinical and functional outcomes) and other implementation outcomes. A cost–utility analysis will estimate incremental costs and quality-adjusted life-years associated with MyPREPED, alongside a secondary cost-effectiveness analysis. Analyses will follow intention-to-treat principles, using mixed-effects models.

**Conclusions:**

This study will provide the first rigorous test of a co-designed, peer-delivered recovery and self-management focused intervention specifically targeting EIPS discharge.

Treating psychosis and associated disorders as early as possible is regarded as best practice in mental health services globally because of the positive impacts this can have on an individual’s clinical and functional outcomes.^1^ The large evidence base for early intervention has led to the establishment of specialist mental health services known as early intervention in psychosis services (EIPS), which provide multidisciplinary, multimodal interventions focused on people aged approximately 12–25 years who are at risk of, or have experienced, a first episode of psychosis. Their goals are to prevent psychosis where possible, reduce the duration of untreated psychosis, restore usual functioning and minimise the impact on families. EIPS are designed to intervene during the ‘critical period’, when treatment is most likely to deliver long-term benefits and when supportive social structures can be mobilised for recovery.^2^

In Australia, EIPS are funded through two parallel systems.^3^ State-funded teams are embedded in local health districts (e.g. 19 teams across 17 sites in New South Wales)^4^ primarily provide care to young people experiencing a first episode of psychosis (FEP). In parallel, federally funded youth mental health services (headspace) operate more than 100 centres nationally; a subset of these centres deliver specialised early psychosis programmes using an Early Psychosis Prevention and Intervention Centre (EPPIC)-aligned model to young people. These programmes support young people with an FEP, who typically receive care for 2–5 years, as well as those identified as being at ultra-high risk of developing psychosis, who are supported for up to 12 months within dedicated ultra-high risk streams. As of 2025, 14 headspace-based early psychosis programmes are operating nationally, with further expansion underway. Across both funding models services typically include medication, psychological therapies, psychoeducation and assistance with education, employment and family support, although local resources determine the scope of delivery.^5–7^ Similar early intervention models for psychosis are implemented internationally, including EIPS in the UK, coordinated specialty care programmes in the USA and established early psychosis services across Europe (e.g. in Scandinavia, The Netherlands, Italy and Spain).

## Transition from EIPS as a critical failure point

Although EIPS have demonstrated significant short-term benefits in clinical and functional outcomes,^1,^ ^2^ transitions in care such as discharge from EIPS, especially into adult or lower-intensity care, remains a key service gap, and may partly be why early gains might be lost over time. Australian epidemiological evidence indicates that young people exit EIPS into markedly different care pathways. In a state-based cohort from Melbourne, 37% of young people were discharged to adult mental health services, whereas 63% transitioned to lower-intensity care, including primary care (46.3%), private psychiatry (11.7%) and private psychology (5.4%).^8^

Qualitative research in Australia highlights how these transitions are experienced by young people and families. Entry into services, staff turnover, hospital admission and discharge, and transition out of EIPS are described as disrupting continuity of care.^4,9^ EIPS discharge is reported as abrupt and undersupported, leaving young people to navigate the adult mental health system alone at a critical stage. Following discharge, young people can enter a period of heightened vulnerability, with increased risks of relapse, readmission and disengagement from care, which can adversely affect long-term mental health, educational, employment and social outcomes.^10^ Internationally, emerging pilot evidence suggests that structured coaching interventions delivered by clinicians to support transition from EIP services may improve functioning outcomes compared with historical controls.^11^

Together, these findings indicate substantial variability in post-EIPS care pathways and suggest that heterogeneity in discharge destinations may contribute to uneven continuity and support during a critical period, underscoring the need for more coordinated transition interventions.

## Peer-supported self-management

Self-management interventions supported by peer support workers (also called peer workers, or as in this trial, peer coaches) offers a potential strategy to address these gaps. Key elements of self-management interventions for serious mental health conditions include psychoeducation, relapse prevention, learning coping strategies, personal recovery and goal-setting, and can also extend to behavioural tailoring to facilitate medication adherence.^12^ Peer support is defined as ‘support or services provided to people with mental health problems by other people who have experienced mental health problems themselves’.^13^ In mental health services, peer support workers constitute one of the fastest-growing components of the mental health workforce internationally.^14^ In Australia, although peer support workers remain a minority relative to the established clinical workforce, the number of paid peer support workers in specialised mental healthcare facilities increased by an average of 18% per year between 2017–2018 and 2021–2022.^15^ This growth rate outpaced that of many traditional professional groups, including psychiatrists, nurses and psychologists. Systematic review evidence of the effectiveness of peer support is mixed, but benefits have been observed for self-reported recovery outcomes.^13,16^ Trial evidence suggests that peer support delivered in conjunction with structured approaches, such as recovery or self-management planning, may be associated with improved outcomes such as reduced readmission rates.^17,18^

The Crisis Resolution Team Optimisation and Relapse Prevention (CORE) randomised controlled superiority trial in the UK demonstrated that at 1-year follow-up, peer-delivered self-management planning following crisis care had significantly lower readmission rates compared with self-management alone (29 *v*. 38%; odds ratio 0.66, 95% CI 0.43–0.99), although the confidence intervals were relatively wide.^18^ The associated trial-based economic evaluation suggested the peer-delivered self-management intervention would likely be cost-effective.^19^ The results point to the potential for peer-delivered self-management planning to reduce readmission rates. Since the CORE trial, the self-management resource has been adapted into a digital tool for use in Australian community mental health services through co-production.^20^

## Need for digital, co-produced solutions

Despite recommendations for use in clinical guidelines^21^ and systematic review evidence of its effectiveness for serious mental illness,^12^ structured self-management planning remains inconsistently implemented in EIPS.^22^ Discharge processes are variable, often clinician-driven and rarely personalised, and support is unable to be sustained after leaving a service. Digital delivery provides a flexible means to extend support beyond clinical contact time, is acceptable and engaging for young people, and can integrate self-management planning into routine during and after discharge.^23^ At the same time, the peer workforce is recognised as a critical enabler of engagement and empowerment in youth mental health services,^24^ yet remains underutilised in supporting transitions out of EIPS.^4^ Further to this, evidence from systematic reviews and emerging studies indicates that digital interventions alone often achieve limited engagement and clinical impact unless complemented by human support,^25–27^ with preliminary evidence suggesting human involvement can enhance adherence, therapeutic alliance and outcomes in digital mental health interventions (including for psychosis spectrum disorders). This reinforces the need for relational contact alongside digital delivery to optimise engagement and effectiveness in youth services.

Overall, these findings highlight the need for structured, personalised and youth-tailored support during transitions out of EIPS. Self-management interventions are associated with improvements in clinical, functional and service use outcomes such as readmission rates, while peer support workers can enhance engagement and promote recovery. Digital delivery offers a flexible mechanism to extend support beyond traditional service boundaries; however, evidence indicates that digital interventions are most effective when embedded within relational models of care that include human support. The My Personal Recovery Plan for Early Discharge (MyPREPED) trial integrates structured self-management planning, peer-led support and digital delivery to address the well-documented gap in transitional care for young people exiting EIPS.

## The MyPREPED trial and development work

**Fig. 1:**
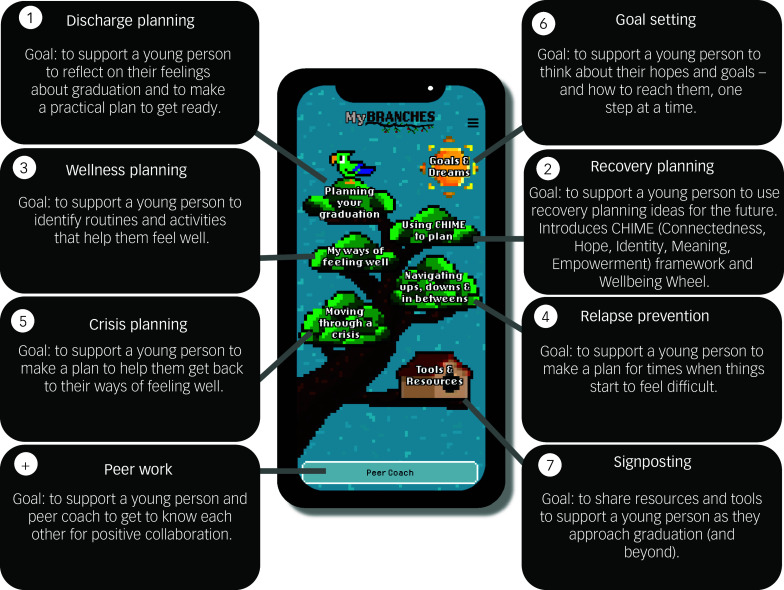
Fig. 1 long description. MyBRANCHES homepage modules and associated goals. MyBRANCHES, My Building Recovery And Navigating Changes with Peer Helpers in Early Intervention Services.

The MyPREPED trial will evaluate a co-designed, digital, peer-delivered self-management intervention co-adapted for young Australians being discharged from EIPS. The trial intervention (MyPREPED) will be delivered by a peer coach, employed in EIPS sites, using the co-designed self-management tool called My Building Recovery and Navigating Changes with Peer Helpers in Early Intervention Services (MyBRANCHES), which will be available via a progressive web app (enabling secure access across smartphones, tablets and desktop devices without requiring installation from commercial app stores to enable more equitable access) or a paper-based version depending on the participant preference. An overview of modules within the MyBRANCHES tool and their associated goals are presented in Fig. 1.

The MyBRANCHES tool has been built through a programme of iterative co-design. A co-designed, paper-based self-management tool, adapted for use in partnership with peer workers in the UK,^28^ was found to be effective at reducing readmission rates in a crisis discharge setting.^18^ More recently, we demonstrated the preliminary acceptability and usability of a co-produced digital version of the self-management tool among adults with severe mental illness, with personalisation, customisation, language, flexibility of use and peer support being key themes.^20^ These studies have informed the current youth version’s development of the tool, renamed through co-design as MyBRANCHES.^29^

Given the limited evidence base for structured, personalised self-management tools in psychosis, particularly those tailored to the discharge transition out of EIPS, there is a need to evaluate not only the clinical effects of the MyPREPED intervention (i.e. up to ten sessions of peer coaching with the MyBRANCHES tool alongside standard discharge care), but also how it can be feasibly and sustainably integrated into routine EIPS delivery. A hybrid type 2 implementation–effectiveness design^30,31^ was therefore selected to enable simultaneous examination of clinical outcomes, implementation processes and contextual determinants influencing real-world uptake. The specific aims of the study are outlined below.

## Aims and research questions

### Aim 1: Experimentally evaluate the effects of MyPREPED versus usual care (control)

We aim to evaluate the impact of the MyPREPED intervention, on mental health recovery alongside other outcomes relating to mental health quality of life, and psychosocial and functional outcomes (see section ‘Outcomes’), by asking the following questions:

- Is MyPREPED more effective than usual care in improving mental health recovery at the primary end-point (12 weeks post baseline)?
- Is MyPREPED more effective than usual care in improving secondary outcomes at the primary end-point (12 weeks post baseline)?
- Are effects maintained at 52 weeks post baseline?

### Aim 2: Evaluate the implementation of MyPREPED in routine EIPS settings

We aim to examine implementation outcomes, including feasibility, acceptability, appropriateness, adoption, fidelity, penetration, retention, sustainability and scalability of MyPREPED, using mixed methods, by asking the following questions:

- Is MyPREPED feasible to implement in real-world EIPS settings, as assessed with the Feasibility of Intervention Measure (FIM) and complementary feasibility indicators?
- Is MyPREPED acceptable and appropriate to young people, peer coaches and clinicians?
- What are the levels of adoption, fidelity, penetration and retention across participating services?
- How usable is the MyBRANCHES tool for young people and peer coaches?
- What implementation barriers, facilitators, and contextual determinants influence sustainability and scalability?
- What practical, operational and contextual lessons can be learned from implementing the trial across sites?

### Aim 3: Evaluate the economic impact of MyPREPED

We aim to evaluate the cost-effectiveness of the MyPREPED intervention relative to usual care, from both health sector and societal perspectives through cost–utility analyses, by asking the following questions:

- Is MyPREPED cost-effective compared with usual care alone from societal and health sector perspectives at 12 weeks post baseline, using utility values (derived from the Recovering Quality of Life 20-item measure (ReQoL-20))?
- Is MyPREPED cost-effective compared with usual care alone from societal and health sector perspectives at 52 weeks post baseline, using utility values derived from the ReQoL-20?
- Is MyPREPED cost-effective compared with usual care alone from societal and health sector perspectives at 12 and 52 weeks post baseline, using utility values derived from the 12-Item Short Form Health Survey (SF-12) as the Short Form 6-Dimension health utility measure (SF-6D)?

## Method

### Frameworks guiding the phased development of the tool, co-production and partnering with consumers

The development of the MyPREPED intervention was guided by a coordinated multi-framework approach to co-production and development of complex interventions,^20^ recognising that no single framework is sufficient for designing, adapting and implementing complex psychosocial interventions in real-world mental health systems.^32^ Consistent with the updated Medical Research Council Framework of developing, testing, evaluating and implementing complex interventions,^33^ the tool was iteratively co-produced through cycles of co-design, feasibility testing and refinement, to ensure clear mechanisms of action and alignment with youth and peer coach needs. Co-production principles shaped all stages of development, embedding lived, learned and practice-based expertise into the content, structure and delivery and evaluation (co-planning, co-design, co-delivery, co-evaluation).^34^ This included co-production of areas such as study design, implementation plans, training and outcomes. A Guidance for Reporting Involvement of Patients and the Public (GRIPP2) short-form^35^ table is provided in Supplementary File 1, outlining the roles of lived-experience partners in the development of the intervention. To maximise implementation potential within routine EIPS, we have selected to use the Consolidated Framework for Implementation Research (CFIR 2.0^36^) to examine the contextual determinants and service readiness, and Proctor’s implementation outcomes taxonomy to guide the specification of constructs such as acceptability, feasibility, fidelity, adoption and sustainability.^37,38^ Further, the MyPREPED trial’s hybrid type 2 design^30^ reflects this integrated approach to testing effectiveness and implementation. Together, this design and complementary frameworks provide a structured and transparent approach to developing, evaluating and interpreting the effectiveness, implementation and scalability of MyBRANCHES within diverse youth psychosis services.

### Design

This is a multi-site, two-arm (1:1), parallel-group, randomised controlled trial of MyPREPED versus standard discharge transition care. Both groups receive standard EIPS care. We will use a hybrid type 2 implementation–effectiveness design aligning with the current Standard Protocol Items: Recommendations for Interventional Trials (SPIRIT) statement^39^ and Consolidated Standards of Reporting Trials (CONSORT) guidance.^40^ This hybrid type 2 trial has co-primary outcomes: implementation feasibility (measured with the FIM)^41^ and recovery (measured with the RAS-DS).^42^

### Setting

The study sites are planned to include eight community-based EIPS located in New South Wales, Victoria and Queensland, Australia. Sites comprise services from four of the federally funded multidisciplinary FEP and ultra-high risk headspace services (Western Sydney, New South Wales; Metro Brisbane area, Queensland) and four state-funded services in metropolitan areas of Sydney (New South Wales), Melbourne (Victoria) and Brisbane (Queensland). Contextual variation across state-funded and federally funded EIPS (e.g. peer workforce maturity, staffing models, discharge processes) will be documented with CFIR-informed site profiles. These contextual data will support interpretation of effectiveness and implementation outcomes in line with SPIRIT guidance.

### Participants

**Fig. 2:**
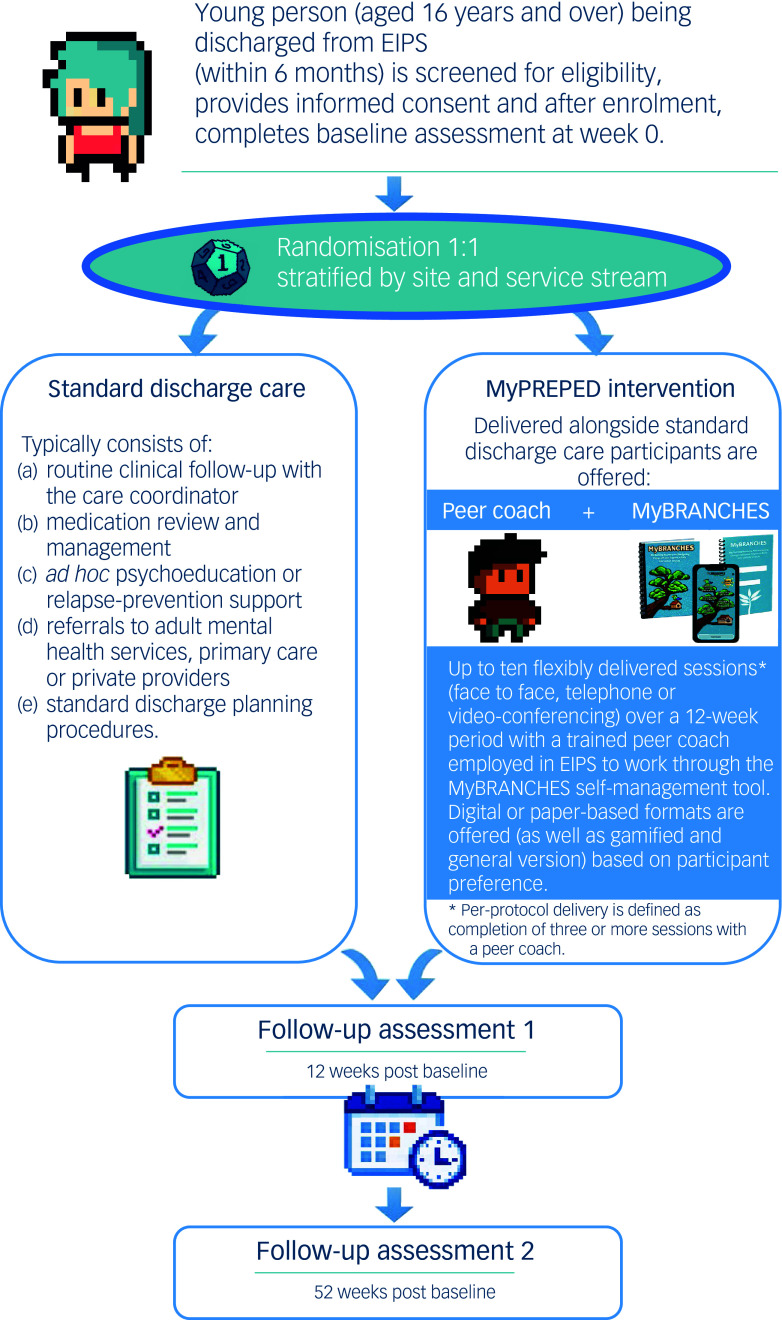
Overview of intervention and participant flow. EIPS, early intervention in psychosis services; MyBRANCHES, My Building Recovery And Navigating Changes with Peer Helpers in Early Intervention Services; MyPREPED, My Personal Recovery Plan for Early Discharge.

Participants must meet the following inclusion criteria: aged 16 years or over, registered with a participating EIPS and approaching discharge (within 6 months), and able to understand English. Exclusion criteria include lack of capacity to consent, as judged by the clinical team; and at high risk that study participation would be unsafe, as judged by the clinical team. Figure 2 presents the planned participant flow through enrolment and allocation, and details the intervention components and follow-up time points. A CONSORT flow diagram is provided in Supplementary File 2.

### Recruitment and consent

Potential participants will be identified by EIPS clinicians as they approach discharge and are referred to the research team. The team will provide study information (via a Participant Information Statement and an e-consent platform) and obtain written or electronic informed consent before conducting baseline assessments (online, via digital conferencing, face to face or via phone per participant preference)^43^ and randomisation. Additional verbal consent before any qualitative interviews will be obtained and formally recorded.

### Randomisation and blinding

Randomisation will be performed centrally with the REDCap randomisation module, with permuted blocks of random size (2, 4) and stratification by site and caseload type (FEP versus ultra-high risk). Allocation will be concealed from outcome assessors and statisticians. Participants and peer coaches cannot be blinded to allocation, but assessors will complete a blinding questionnaire post intervention, and any suspected breaches will be documented for sensitivity analyses.

### Pilot

Before full implementation, a small pilot phase will be undertaken to assess the feasibility and acceptability of recruitment, intervention delivery and study procedures within participating EIPS settings, with pilot participants rolled into the full trial sample unless major feasibility or safety concerns are identified.

### Intervention: MyPREPED

The MyPREPED intervention is described in accordance with the Template for Intervention Description and Replication (TIDieR) checklist^44^ to support replication (Table 1). MyPREPED is a complex, peer-delivered, self-management and discharge-planning intervention delivered by trained lived-experience peer coaches, using the MyBRANCHES self-management tool. MyPREPED comprises two integrated components: (a) structured peer-coaching sessions and (b) guided use of the MyBRANCHES tool, delivered in digital or paper-based format according to participant preference.

**Table 1:** TIDieR checklist for MyPREPED Table 1 long description.

TIDieR item	Description of MyPREPED intervention
1. Brief name	MyPREPED, a peer-delivered self-management and discharge-planning intervention for young people transitioning from EIPS. The self-management tool used is called MyBRANCHES. MyPREPED comprises the combined delivery of peer-coaching sessions and guided use of the MyBRANCHES self-management tool.
2. Why (rationale)	Designed to improve discharge outcomes by providing structured self-management, personalised recovery planning and lived-experience support during the transition out of EIPS.
3. What (materials)	Materials include the MyBRANCHES tool (digital and paper-based options available), peer coach manual, fidelity checklists, training modules (2-day workshop online content), supervision guides and logic model. The digital component is a PWA, which uses responsive design allowing flexible access and device-agnostic use, supporting implementation for diverse preferences. The MyBRANCHES tool is versioned, with updates documented across the trial period, and participants retain access to their materials following discharge. The digital tool is gently gamified (avatar, customisation options) and allows optional sharing with families/clinicians. All materials will be openly available via the OSF.
4. What (procedures)	Sessions follow a standard structure: brief check-in, collaborative selection of MyBRANCHES modules, guided completion of modules and agreement on next steps. Modules include discharge planning, recovery planning, well-being planning, relapse-prevention planning, crisis planning, goal-setting, coping strategies, psychoeducation and lived-experience examples. Peer coaches document session content, delivery mode and duration in logbooks. Any safety concerns identified during sessions are managed in line with prespecified risk escalation protocols and communicated to the research and clinical care team as required. Fidelity is monitored through checklists and supervision review.
5. Who provides	Delivered by trained lived-experience peer workers (‘peer coaches’) employed by participating services. The term for their role here was chosen as ‘peer coaches’ by the lived experience advisory group. Peer coaches typically hold a Certificate IV in Mental Health (or equivalent), have experience in psychosis/mental health settings, and complete formal MyPREPED training and safety procedures. Ongoing competency is supported through monthly supervision and review of session checklists for fidelity. Monthly group supervision is provided by study investigators.
6. How (mode of delivery)	One-to-one peer-coaching sessions delivered face to face, by telephone or via telehealth. Participants may use digital or paper versions of MyBRANCHES. Engagement strategies include flexible scheduling, multimodal delivery and brief between-session check-ins.
7. Where	Delivered in routine EIPS settings or remotely (telephone/telehealth) depending on participant preference and service context.
8. When and how much (dose)	Up to ten sessions of approximately 45–60 min each. Minimum effective dose defined as three or more sessions for per-protocol analyses. Sessions generally commence within 12 weeks before anticipated discharge, with timing adjusted based on clinical trajectory and participant preference.
9. Tailoring	Tailoring occurs jointly between peer coach and participant. Elements tailored include module prioritisation, pacing, scheduling, delivery format (digital or paper), modality (in person/telephone/video-conferencing), session frequency (weekly/fortnightly/tapering) and involvement of family/clinicians. Content and delivery are also tailored youth preferences (e.g. access to a non-gamified version).
10. Modifications	Any adaptations during feasibility testing will be documented following CONSORT-SPI and SPIRIT guidelines, with approval by the study governance group and documentation of rationale.
11. How well (planned fidelity assessment)	Fidelity monitored through peer coach logbooks, structured fidelity checklists and monthly supervision reviews. Fidelity assessments are conducted by trained members of the research team. Deviations and contextual adaptations will be recorded.
12. How well (actual fidelity)	To be reported post trial, including adherence to core components, dose delivered and received, mode of delivery and participant engagement patterns.
Fixed (prespecified) components	Core MyBRANCHES modules (discharge planning, recovery planning, well-being planning, relapse-prevention, crisis planning, and goal-setting); structured session flow (check-in, module selection, guided use, next steps); standardised training package; peer coach role requirements; fidelity monitoring procedures; session documentation requirements; maximum dose (up to ten sessions); core safety and risk-management procedures; availability of digital and paper materials; open-science access to manuals and tools.
Adaptable (flexible) components	Module prioritisation based on participant goals; mode of delivery (in person/telephone/telehealth); format (digital or paper workbook); session frequency (weekly/fortnightly/tapering); scheduling and pacing; optional between-session check-ins; extent of family/clinician involvement; commencement timing relative to discharge; culturally or contextually relevant examples used by peer coaches; tailoring to local service workflows and participant preferences.

TIDieR, Template for Intervention Description and Replication; MyPREPED, My Personal Recovery Plan for Early Discharge; MyBRANCHES, My Building Recovery And Navigating Changes with Peer Helpers in Early Intervention Services; EIPS, early intervention in psychosis services; PWA, Progressive Web Application; OSF, Open Science Framework; CONSORT-SPI, Consolidated Standards of Reporting Trials – Social and Psychological Interventions; SPIRIT, Standard Protocol Items: Recommendations for Interventional Trials.

MyPREPED includes both fixed (prespecified core components) and flexible (personalised) elements, which are fully detailed in Table 1. A logic model and description of active ingredients are provided in Supplementary File 3. Content overview and interface are provided in Supplementary Files 4 and 5, respectively.

### Control: usual care

Participants in the control arm will receive EIPS usual care and standard discharge planning, with no structured peer-coaching sessions using MyBRANCHES provided. Services may provide variable psychoeducation and support (including peer support); this will be documented descriptively in implementation logs. Treatment as usual typically includes routine clinical follow-up with the primary clinician/care coordinator; medication review and management; *ad hoc* psychoeducation or relapse-prevention support; referrals to adult mental health services, primary care or private providers; and standard discharge-planning procedures. Treatment as usual varies across services, so site-level descriptions of standard care and discharge processes will be documented to support interpretation and reproducibility.

### Minimising contamination

Several strategies will be used to minimise contamination between trial arms. Peer coaches delivering the MyPREPED intervention will deliver the intervention only to participants randomised to the intervention arm. Although peer coaches may be employed directly through trial funding or embedded within participating services depending on local arrangements, their MyPREPED role will be distinct from any peer support provided as part of usual care, and they will not provide intervention content to control participants. Where peer support is available as part of usual care within some of the participating services, peer workers not involved in MyPREPED delivery will not receive training in MyPREPED procedures or access to the MyBRANCHES tool during the trial period. Intervention materials, including access to the MyBRANCHES progressive web application, paper workbooks and peer-coaching guidance, will be restricted to intervention participants. Services will be asked not to use MyBRANCHES in routine practice until trial completion. Any incidental exposure or potential contamination reported by participants, peer coaches or clinicians will be documented through session logs and qualitative interviews, and examined in sensitivity and process analyses.

### Outcomes

The trial will include a comprehensive set of implementation, effectiveness and economic outcomes assessed across multiple time points. Consistent with the study’s co-produced design, lived experience researchers, peer coaches, young people and transdisciplinary academic staff were involved in shaping outcome selection and prioritisation. For example, the primary effectiveness recovery outcome – RAS-DS score^45^ at 12 weeks – was co-selected with lived-experience partners, who identified its strong conceptual alignment with the MyPREPED content domains and its relevance to youth-led definitions of recovery. RAS-DS outcomes will be measured at baseline, 12 weeks post baseline (primary end-point) and 52 weeks post baseline. Secondary effectiveness outcomes include mental health-related quality of life (ReQoL-20),^46^ psychological distress (Kessler-10),^47^ well-being (My Life Tracker-5),^48^ general self-efficacy (Short-Form General Self-Efficacy Scale),^49^ social inclusion (Filia Social Inclusion Measure),^50^ service satisfaction (Client Satisfaction Questionnaire-8),^51^ health literacy (Single-Item Literacy Screener)^52^ and clinician-rated functioning (Social and Occupational Functioning Assessment Scale);^53^ general health-related functioning and quality of life will also be assessed for economic evaluation purposes only, using the SF-12.^54^ The primary implementation outcome is feasibility, which will be measured using the FIM^41^ at 12 weeks among participants in the intervention arm and peer coaches. Additional implementation outcomes comprise acceptability (Acceptability of Intervention Measure^41^); appropriateness (Intervention Appropriateness Measure^41^); adoption, fidelity, penetration, retention and usability (System Usability Scale^55^); safety, and indicators of sustainability and scalability, informed by the Intervention Scalability Assessment Tool framework. The economic evaluation will use a micro-costing approach to estimate the cost of the MyPREPED intervention. The purpose-built Resource Use Questionnaire (RUQ^56^) will measure participants’ use of health services and lost productivity, which will be monetised by attaching standard Australian unit costs. Outcomes for the economic evaluation will be utility values and quality-adjusted life-years derived from the Recovering Quality of Life – Utility Index (ReQoL-UI) and Short Form 6 Dimensions (SF-6D). A consolidated schedule of measures, summarising timing and instruments across all outcome domains, is presented in Table 2.

**Table 2:** Outcome measures of the study Table 2 long description.

Outcome category	Item	Baseline	Post-intervention (12 weeks)	Follow-up (52 weeks)
Primary effectiveness outcome	Recovery Assessment Scale-DS (RAS-DS)^44^	✓	✓	✓
Secondary effectiveness outcomes	Recovering Quality of Life (ReQoL-20)^a 47^	✓	✓	✓
	Short-Form General Self-Efficacy Scale (GSE-6)^50^	✓	✓	✓
	My Life Tracker (MLT-5)^49^	✓	✓	✓
	Filia Social Inclusion Measure (F-SIM-16)^51^	✓	✓	✓
	Client Satisfaction Questionnaire-8 (CSQ-8)^46,52^	✓	✓	
	Short Form Health Survey (SF-12)^a 55^	✓	✓	✓
	Single-Item Literacy Screener (SILS) (ref)^53^	✓	✓	✓
	Kessler-10 (K10)^48^	✓	✓	✓
	Social and Occupational Functioning Assessment Scale (SOFAS) – clinician-reported^54^	✓	✓	
Primary implementation outcome	Feasibility of Intervention Measure (FIM)^42^		✓	
Secondary implementation outcome	Acceptability of Intervention Measure (AIM)^42^		✓	
	Intervention Appropriateness Measure (IAM)^42^		✓	
	Administrative metrics	✓	✓	✓
	System Usability Scale (SUS)^56^		✓	
	Qualitative interviews		✓	
Economic outcomes	Brief Resource Use Questionnaire (RUQ) service use (e.g. emergency department presentations, hospital admissions, crisis service use, other service use)^57^	✓	✓	✓
	Medication: Brief Resource Use Questionnaire (RUQ) medications list – clinician-reported,^b 57^ and client-reported^c^	✓^b^	✓^b^	✓^c^
Safety outcomes	Adverse events; qualitative reports on distress		✓	✓

a. Economic outcomes.

b. Clinician-reported.

c. Client-reported.

### Sample size and power

The total planned sample size is 260 participants (130 per arm). This sample size aligns with the original calculation outlined in the protocol and statistical analysis plan, and was based on detecting a small-to-moderate effect (seven-point difference in RAS-DS score, s.d. = 15, Cohen’s *d* = 0.47) on the primary effectiveness outcome while ensuring adequate precision for feasibility and implementation estimates. We assumed a seven-point between-group difference on the RAS-DS at 12 weeks, corresponding to a small-to-moderate effect size (Cohen’s *d* of approximately 0.47) using a conservative s.d. of 15. This assumption was informed by prior RAS-DS psychometric studies in Australian community mental health samples, which report somewhat smaller standard deviations,^42,57^ and was chosen to ensure adequate power for effectiveness estimates. The sample size calculation also assumes up to 20% attrition, an intracluster correlation coefficient of 0.02 (consistent with published methodological guidance showing intracluster correlation coefficients of 0.01–0.05 for patient-reported mental health outcomes^58^) and an average cluster size of 20, with parameter optimisation undertaken to confirm feasibility under these assumptions. Full details of the sample size modelling and assumptions are provided in the statistical analysis plan (available via the Australian and New Zealand Clinical Trial Registry, under identifier ACTRN12626000169347).

### Statistical analysis

All analyses will follow the intention-to-treat principle, with a complementary per-protocol sensitivity set defined *a priori* in the statistical analysis plan. Continuous outcomes, including the primary effectiveness measure (RAS-DS) and secondary measures, will be analysed using linear mixed-effects models with fixed effects for treatment group, time and the group×time interaction, site and caseload group (FEP versus ultra-high risk) to reflect the stratified randomisation. Random intercepts for participants will account for within-participant correlation. A sensitivity analysis will examine a model including a random intercept for site if sufficient numbers per site permit stable estimation; sites recruiting no participants will drop out of the factor, and sites recruiting fewer than five participants will remain in the main analysis, but will be excluded in an additional sensitivity analysis. Consistent with the addendum to the International Council for Harmonisation (ICH) E9 harmonised guideline on statistical principles for clinical trials, ICH E9(R1), the handling of intercurrent events and missing data will follow the estimand framework prespecified in the statistical analysis plan.^59,60^ For the primary effectiveness estimand, we will apply a hybrid approach that recognises both participants’ capacity to continue treatment and the practical constraints that may limit their ability to do so. In this method, intercurrent events related to treatment tolerability (e.g. withdrawal owing to adverse effects) will be handled with a treatment-policy strategy (intention to treat) and events related to contextual or service factors (e.g. service disruption) will be handled using a hypothetical strategy, treating outcomes as if treatment had continued, implemented via multiple imputation after removing outcome observations post intercurrent events (ICE).^61–63^ We will conduct a range of sensitivity and exploratory analyses, including (a) a treatment policy estimator, using multiple imputation for all missing data; (b) a conservative estimator, using baseline-observation-carried-forward for missing 12-week outcomes; (c) a sensitivity analysis excluding sites with very low recruitment and (d) exploratory analyses of potential dose–response relationships. Full analytical specifications, including model diagnostics and estimand definitions, are detailed in the statistical analysis plan.

### Qualitative and mixed-methods analysis

Qualitative data from interviews and focus groups with patients, peer coaches and clinical staff will be analysed with a combination of reflexive thematic analysis^64,65^ and framework analysis focused on implementation outcomes.^66^ Reflexive thematic analysis will be used to generate inductive, interpretive insights, following an iterative approach consistent with contemporary qualitative methodology. The topic guides were explicitly informed by CFIR 2.0^67^ to capture multi-level determinants of implementation, and by Proctor’s implementation outcomes framework to examine constructs such as acceptability, feasibility, appropriateness, adoption and sustainability.^37,38^ Framework analysis will then be used to systematically map emergent themes onto these implementation constructs and across exploration, preparation, implementation and sustainment phases. Coding will proceed with a combination of inductive and deductive approaches, allowing both theoretically informed constructs and novel, data-driven insights to emerge. Findings from the qualitative analysis will be integrated with quantitative implementation outcomes (e.g. see Arnautovska et al, 2026),^68,69^ using a convergent parallel design. This will enable a mixed-methods interpretation that examines cases where implementation experiences diverge from expected patterns and identifies factors shaping the feasibility, acceptability and scalability of MyPREPED across diverse EIPS contexts.

### Economic evaluation

The economic evaluation will comprise a within-trial cost–utility analysis conducted from both societal and health sector perspectives at 12 and 52 weeks. The primary economic analysis will adopt a societal perspective at 12 weeks, and compare MyPREPED plus usual care against usual care alone. Costs will include the cost of developing, delivering and maintaining MyPREPED, healthcare costs borne by the government, participants’ out-of-pocket costs and productivity losses (absenteeism and presenteeism), measured via a purpose-built Resource Use Questionnaire. Health outcomes will be expressed as quality-adjusted life-years derived from the ReQoL-20, using the Australian value set, with secondary analyses using SF-6D utilities derived from the SF-12. Total costs and quality-adjusted life-years will be compared between arms, and incremental cost–utility ratios will be estimated at 12 and 52 weeks, with uncertainty summarised by non-parametric bootstrapping and cost-effectiveness acceptability curves. Secondary analyses will adopt a health-sector perspective and explore alternative assumptions and utility measures, as detailed in the statistical analysis plan.

### Reporting

Reporting will conform with the SPIRIT 2025 Statement^39^ and CONSORT 2025^40^ guidelines, with implementation reporting informed by the Standards for Reporting Implementation statement for implementation studies.^70^ Qualitative components will be reported in line with consolidated criteria for reporting qualitative research (COREQ) guidance,^71^ and mixed-methods integration will be informed by Good Reporting of A Mixed Methods Study (GRAMMS) recommendations.^72^ The economic evaluation will follow international best practice and be reported in accordance with the Consolidated Health Economic Evaluation Reporting Standards (CHEERS) 2022 statement.^73^ Patient and public involvement will be reported following GRIPP2 short-form guidance,^35^ detailing roles of young people, peer coaches and lived-experience advisors across design, outcome selection and interpretation. We will use updated versions of recommendations, statements, checklists and guidelines where appropriate.

### Ethics, safety and data management

The trial has received ethical approval from the relevant Human Research Ethics Committees (approval numbers: X25-0057; 2025/ETH00610) and will be conducted in accordance with the National Statement on Ethical Conduct in Human Research, the International Council for Harmonisation Good Clinical Practice guidelines and the principles of the Declaration of Helsinki. Safety oversight will be provided by the Trial Management Group, which monitors adherence to standardised definitions and reporting procedures for adverse events and serious adverse events, including predefined quantitative thresholds that reflect the trial’s focus on safety as an implementation outcome. Given the minimal-risk nature of the intervention, a formal Data Safety Monitoring Board is not required; oversight will be provided by the Trial Management Group. All study data will be managed in accordance with institutional policies and the project’s Open Science and Data Sharing plan, aligned with SPIRIT 2025 and CONSORT 2025 guidance. This includes the use of secure, access-controlled data environments, rigorous confidentiality protections, de-identification procedures before data release and a controlled-access model for sharing de-identified participant data-sets, analysis code and replication materials following publication of co-primary outcomes. The trial is among the first in Australia’s mental health sector to be developed in accordance with SPIRIT 2025, and trial conduct and reporting will adhere to CONSORT 2025 guidance including explicit open science planning, structured reporting of implementation outcomes and preregistration of analytic decisions.

### Protocol version and amendments

This is protocol version v2 (24 November 2025). Future amendments will be submitted to the Human Research Ethics Committee (HREC) and updated in the trial registry. Revisions to the statistical analysis plan will also be documented and updated on open science data repositories. All documentation and de-identified data emanating from the project will be housed on University of Sydney Figshare (or an institutionally endorsed equivalent) following publication of the main trial findings. Meta-data will be publicly visible, but data will be made available upon application, approval and agreement to a data use agreement.

## Discussion

### Innovation

This trial represents an important methodological and translational innovation as the first hybrid type 2 study to evaluate a novel, co-produced, peer-delivered self-management intervention at the point of discharge from EIPS. MyPREPED brings together structured, evidence-informed self-management content, peer coaching and flexible digital and paper-based delivery options. Grounded in lived-experience leadership and participatory design, the intervention is expected to enhance personal recovery, well-being and quality of life by strengthening young people’s self-management capabilities during a period often marked by uncertainty. Peer coaching is anticipated to play a critical role in supporting engagement, normalising recovery-oriented practices and helping young people translate strategies into day-to-day contexts. We will also capture through qualitative interviews any indicative benefits or challenges from the perspective of the peer coaches.

From an implementation perspective, this trial also provides a rigorous examination of feasibility, acceptability, fidelity, appropriateness, adoption and scalability within routine EIPS settings, generating comprehensive mixed-methods evidence to inform national roll-out and possible adaptations to implementation across any other contexts.

The embedded economic evaluation extends the contribution of this work by determining the cost-effectiveness and resource implications of integrating peer-delivered self-management into routine transitional care for young people exiting EIPS. Previous trials have suggested the economic potential of related approaches. For example, the CORE trial reported the potential cost-effectiveness for peer-delivered self-management following crisis care in the UK,^19^ and the Early signs Monitoring to Prevent relapse in psychosis and prOmote Well-being, Engagement and Recovery (EMPOWER) feasibility trial showed favourable cost-effectiveness for a blended digital and peer-delivered relapse-prevention intervention.^74^ However, economic evidence specific to peer-delivered self-management at the point of EIPS discharge, particularly within the Australian youth mental health context, remains limited. The economic evaluation embedded within MyPREPED will therefore address this gap, providing decision makers with contextually relevant information to support commissioning and scale-up.

Methodologically, the trial is among the first in Australia’s mental health sector to adhere with CONSORT 2025 and SPIRIT 2025 guidance, building on co-design and consumer engagement, ensuring transparent reporting, open science practices and rigorous specification of implementation outcomes.

### Limitations and future directions

The study has several limitations. Recruitment is currently limited to English-speaking participants, which may constrain generalisability to culturally and linguistically diverse populations. Usual care practices vary across sites, introducing contextual heterogeneity that may influence comparative outcomes. The trial has feasibility-level power for some secondary and mediation analyses, and findings should therefore be interpreted with appropriate caution. Finally, the study does not include routine medical record or administrative data linkage because of the complexity of harmonising data access across multiple EIPS, services and jurisdictions, which may limit objective capture of some areas of interest such as hospital admission outcomes. Despite these limitations, the trial is well-positioned to generate high-quality evidence on both the clinical and implementation value of peer-delivered self-management during a critical transition point in the early psychosis care continuum.

To summarise, the MyPREPED trial will generate integrated evidence on the feasibility, acceptability, clinical benefit and cost-effectiveness of a co-produced, peer-delivered self-management intervention delivered at the point of discharge from EIPS. The findings will inform future implementation and potential scale-up of MyPREPED within youth mental health systems, addressing a longstanding gap in transitional care. Results will be disseminated through peer-reviewed publications, conferences and partnerships with services and lived-experience stakeholders.

### Lived-experience commentary

Our lived-experience voices are woven into each developmental stage of MyPREPED trial and MyBRANCHES tool. The trial developed from a co-designed process where feedback from young people and peer coaches in EIPS guided the language, usability and accessibility of the tool. We believe the tool fills an innovation gap where research-backed and co-designed tools can support mental health service delivery and the historical role of peer coaches as change makers in the industry. Below are some comments from peer coaches assisting in the delivery of this tool:

‘This project genuinely framed our lived experiences as expertise, allowing us to fully lead with the guidance of mental health researchers. This ultimately supports young people at a broader level.’ Sam Batara (Youth Peer Support Coach)

‘My transition out of EIPS was an abrupt and overwhelming experience. Despite the transitioning out is a high-risk period for young people, it’s shocking how few research-based mental health applications exist for this stage. I’m honoured to contribute to MyBRANCHES for how it targets this industrial gap and while honouring the needs expressed by young people.’ Simran Singh (Youth Peer Support Coach)

From our perspective, the project shows the way evidence and lived experience-based tools could be used with pre-existing services to promote innovation in peer support service delivery. Although MyBRANCHES targets the transition out of EIPS, we think more tools should be developed to support each stage experienced in EIPS. With more tools targeting the needs of young people, peer coaches will be armed with more resources to encourage the confidence, hope and autonomy that young people can have over their recovery journey.

## Supporting information

10.1192/bjo.2026.12056.sm001 Milton et al. supplementary material Milton et al. supplementary material

## Data Availability

Per the study’s statical analysis plan details, de-identified participant data, analytic code and supporting materials will be made available following publication of the co-primary outcomes via the University of Sydney Figshare repository (or institutionally endorsed equivalent), subject to approval of a data access request and data use agreement.

## Supplementary material

The supplementary material is available online at https://doi.org/10.1192/bjo.2026.12056

## Fig. 1 Long description

Panel 1: Discharge Planning. Goal: to support a young person to reflect on their feelings about graduation and to make a practical plan to get ready. Panel 2: Recovery Planning. Goal: to support a young person to use recovery planning ideas for the future. Introduces CHIME (Connectedness, Hope, Identity, Meaning, Empowerment) framework and Wellbeing Wheel. Panel 3: Wellness Planning. Goal: to support a young person to identify routines and activities that help them feel well. Panel 4: Peer Work. Goal: to support a young person and peer coach to get to know each other for positive collaboration. Panel 5: Crisis Planning. Goal: to support a young person to make a plan to help them get back to their ways of feeling well. Panel 6: Goal Setting. Goal: to support a young person to think about their hopes and goals and how to reach them, one step at a time. Panel 7: Signposting. Goal: to share resources and tools to support a young person as they approach graduation (and beyond).

Navigate back to Fig. 1.

## Table 1 Long description

The table presents the TIDieR checklist for the MyPREPED intervention, detailing various aspects of the intervention. It has 12 rows and 2 columns. The first column lists the TIDieR items, and the second column describes the corresponding aspects of the MyPREPED intervention. Row 1: TIDieR Item, Description of MYPREPED intervention. Row 2: 1. Brief name, MyPREPED, a peer-delivered self-management and discharge-planning intervention for young people transitioning from EIPS. MyPREPED comprises the combined delivery of peer-coaching sessions and guided use of the MYBRANCHES self-management tool. Row 3: 2. Why (rationale), Designed to improve discharge outcomes by providing structured self-management, personalized recovery planning and lived-experience support during the transition out of EIPS. Row 4: 3. What (materials), Materials include the MYBRANCHES tool (digital and paper-based options available), peer coach manual, fidelity checklists, training modules 2-day workshop online content, supervision guides and logic model. The digital component is a PWA, which uses responsive design allowing flexible access and device-agnostic use, supporting implementation for diverse preferences. The MYBRANCHES tool is versioned, with updates documented across the trial period, and participants retain access to their materials following discharge. The digital tool is gently gamified (avatar, customization options) and also includes optional sharing with families/clinicians. All materials will be openly available via the OSF. Row 5: 4. What (procedures), Sessions follow a standard structure: brief check-in, collaborative selection of MYBRANCHES modules, guided completion of modules and agreement on next steps. Modules include discharge planning, recovery planning, well-being planning, relapse prevention planning, crisis planning, goal-setting, coping strategies, psych education and lived-experience examples. Peer coaches document session content, delivery mode and duration in rosters. Any safety concerns identified during sessions are managed in line with prespecified risk escalation protocols and communicated to the research and clinical care team as required. Fidelity is monitored through checklists and supervision review. Row 6: 5. Who provides, Delivered by trained lived-experience peer workers (peer coaches) employed by participating services. The term for their role here was chosen as peer coaches by the lived experience advisory group. Peer coaches typically hold a Certificate IV in Mental Health (or equivalent), have experience in psychosocial/mental health settings, and complete formal MYPREPED training and safety procedures. Ongoing competency is supported through monthly supervision and review of session checklists for fidelity. Monthly group supervision is provided by study investigators. Row 7: 6. How (mode of delivery), One-to-one peer-coaching sessions delivered face to face, by telephone or via telehealth. Participants may use digital or paper versions of MYBRANCHES. Engagement strategies include flexible scheduling, mutual goals and brief between-session check-ins. Row 8: 7. Where, Delivered in routine EPIS settings or remotely telephone/telehealth depending on participant preference and service context. Row 9: 8. How often and how much (dose), Up to ten sessions of approximately 45-60 min each. Minimum effective dose defined as three or more sessions for protocol analyses. Sessions generally commence within 12 weeks before anticipated discharge, with timing adjusted based on clinical trajectory and participant preference. Row 10: 9. Tailoring, Tailoring occurs jointly between peer coach and participant. Elements tailored include module prioritization, pacing, scheduling, delivery format (digital or paper), modality (in person/telephone/video-conferencing), session frequency (weekly/fortnightly/tapering) and involvement of family/clinicians. Content and delivery are also tailored to youth preferences (e.g. access to a non-gamified version). Row 11: 10. Modifications, Any adaptations will be feasibility testing will be documented following CONSORT-SPI and SPIRIT guidelines, with approval by the study governance group and documentation of rationale. Row 12: 11. How well (planned fidelity assessment), Fidelity monitored through peer coach logbooks, structured fidelity CHECKLISTS and monthly supervision reviews. Fidelity assessments are conducted by trained members of the research team. Deviations and contextual adaptations will be recorded. Row 13: 12. How well (actual fidelity), To be reported post trial, including adherence to core components, dose delivered and received, mode of delivery and participant engagement patterns. Fixed (prespecified) components: Core MYBRANCHES modules (discharge planning, recovery planning, well-being planning, relapse-prevention, crisis planning and goal-setting), structured session flow (check-in, module selection, guided use, next steps), standardized training package, peer coach log requirements, fidelity monitoring procedures, session duration requirements, maximum dose (up to ten sessions), core safety and risk-management procedures, availability of digital and paper materials, open-science access to manuals and tools. Adaptable (flexible) components: Module prioritization based on participant goals, mode of delivery (in person/telephone/telehealth), format (digital or paper workbooks), session frequency (weekly/fortnightly/tapering), scheduling and pacing, optional between-session check-ins, extent of family/clinician involvement, commencement timing relative to discharge, culturally and contextually relevant examples used by peer coaches, tailoring to local service workflows and participant preferences.

Navigate back to Table 1.

## Table 2 Long description

A table summarizing the schedule of measures for a study, including various outcomes assessed at different time points. The table has 20 rows and 4 columns. The columns are labeled Item, Baseline, Post-intervention (12 weeks), and Follow-up (52 weeks). The rows are categorized into Primary effectiveness outcome, Secondary effectiveness outcomes, Primary implementation outcome, Secondary implementation outcomes, Economic outcomes, and Safety outcomes. Each row lists specific measures and their assessment times with checkmarks indicating when each measure is taken. Row 1: Recovery Assessment Scale-DS (RAS-DS), Baseline: checked, Post-intervention (12 weeks): checked, Follow-up (52 weeks): checked. Row 2: Recovering Quality of Life (ReQoL-20), Baseline: checked, Post-intervention (12 weeks): checked, Follow-up (52 weeks): checked. Row 3: Short-Form General Self-Efficacy Scale (GSE-6), Baseline: checked, Post-intervention (12 weeks): checked, Follow-up (52 weeks): checked. Row 4: My Life Tracker (MLT-5), Baseline: checked, Post-intervention (12 weeks): checked, Follow-up (52 weeks): checked. Row 5: Filia Social Inclusion Measure (F-SIM-16), Baseline: checked, Post-intervention (12 weeks): checked, Follow-up (52 weeks): checked. Row 6: Client Satisfaction Questionnaire-8 (CSQ-8), Baseline: checked, Post-intervention (12 weeks): checked, Follow-up (52 weeks): checked. Row 7: Short Form Health Survey (SF-12), Baseline: checked, Post-intervention (12 weeks): checked, Follow-up (52 weeks): checked. Row 8: Single-item Literacy Screener (SILS), Baseline: checked, Post-intervention (12 weeks): checked, Follow-up (52 weeks): checked. Row 9: Kessler-10 (K10), Baseline: checked, Post-intervention (12 weeks): checked, Follow-up (52 weeks): checked. Row 10: Social and Occupational Functioning Assessment Scale (SOFAS) – clinician-reported, Baseline: checked, Post-intervention (12 weeks): checked, Follow-up (52 weeks): checked. Row 11: Feasibility of Intervention Measure (FIM), Baseline: unchecked, Post-intervention (12 weeks): checked, Follow-up (52 weeks): unchecked. Row 12: Acceptability of Intervention Measure (AIM), Baseline: unchecked, Post-intervention (12 weeks): checked, Follow-up (52 weeks): unchecked. Row 13: Intervention Appropriateness Measure (IAM), Baseline: unchecked, Post-intervention (12 weeks): checked, Follow-up (52 weeks): unchecked. Row 14: Administrative metrics, Baseline: unchecked, Post-intervention (12 weeks): checked, Follow-up (52 weeks): unchecked. Row 15: System Usability Scale (SUS), Baseline: unchecked, Post-intervention (12 weeks): checked, Follow-up (52 weeks): unchecked. Row 16: Qualitative interviews, Baseline: unchecked, Post-intervention (12 weeks): unchecked, Follow-up (52 weeks): checked. Row 17: Brief Resource Use Questionnaire (RUQ) service use, Baseline: checked, Post-intervention (12 weeks): checked, Follow-up (52 weeks): checked. Row 18: Medication: Brief Resource Use Questionnaire (RUQ) medications list – clinician-reported, Baseline: checked, Post-intervention (12 weeks): checked, Follow-up (52 weeks): checked. Row 19: Medication: Brief Resource Use Questionnaire (RUQ) medications list – client-reported, Baseline: checked, Post-intervention (12 weeks): checked, Follow-up (52 weeks): checked. Row 20: Adverse events, qualitative reports on distress, Baseline: checked, Post-intervention (12 weeks): checked, Follow-up (52 weeks): checked.

Navigate back to Table 2.
