# Supplementary material for: Protocol for the MyPREPED trial: hybrid type 2 effectiveness–implementation trial of a peer-delivered digital self-management tool (MyBRANCHES) for young people in Australia transitioning from early intervention in psychosis services
Source: BJPsych Open. 2026 Jul 27;12(4):e200. doi: 10.1192/bjo.2026.12056 (PMC13419609; doi:10.1192/bjo.2026.12056)
Supplement: Milton et al. supplementary material [file S2056472426120560sup001.docx]

**Supplementary Table 1. GRIPP2 for pre-trial work with MyPREP-ED**

| **GRIPP2-SF Item** | **Description of PPI Activity in MyPREP-ED co-production** |
| --- | --- |
| 1. Aim of PPI | To co-produce the MyPREP-ED intervention and trial procedures with people with lived and living experience of psychosis and service transition, ensuring acceptability, relevance, safety, and youth-centred design. |
|  | - LE representation within the project leadership team, contributing to strategic decisions, governance, and interpretation of emerging findings.  - A Lived Experience (LE) Advisory Group of young people and peer workers (ongoing). - Co-design workshops with young people, peer workers, clinicians and carers.  - Iterative user-testing of MyBRANCHES content and digital features with co-designers .  - Ongoing consultation with peer workforce representatives to refine training, supervision, and safety processes. |
| 3. Results of PPI (influence on study) | - The term “peer coach” was selected by LE contributors and reflects preferred role framing.  - Language, content, tone, layout, design aesthetic and sequencing of MyBRANCHES modules shaped by young people and LE advisors.  - Inclusion of lived-experience reflections, coping strategies, and youth-friendly examples.  - Decision to provide both digital and paper-based formats and refinements to digital gamification (avatar, customisation, language choice).  - Peer-coach training package strengthened with recovery-orientation, collaboration, and trauma-informed practice.  - LE input informed flexibility parameters (frequency, modality, family involvement).  - The risk-management pathway was refined to support psychological safety and peer-coach confidence.  - Peers shared insights about strategies to support practical implementation of MyPREP-ED at trial. |
| 4. Reflections / critical perspective | PPI strengthened contextual relevance, acceptability, and feasibility. Having LE representation within governance enhanced accountability to lived experience throughout development. Challenges included ensuring that diverse preferences were surfaced and accommodated (where feasible). For example, careful critical consideration was required to balance the need for MyBRANCHES and the MyPREP-ED intervention to accommodate personal choice and flexibility, while also maintaining fidelity, and supporting greater levels of engagement across certain sites. |
| 5. Future plans for PPI | - Continued engagement of the LE Advisory Group throughout trial.  - Ongoing LE representation within the leadership team to guide decision-making, adaptation, and interpretation.  - LE review of training materials, fidelity tools, and participant-facing documents.  - LE involvement in interpreting qualitative data.  - Collaboration with LE contributors to co-develop accessible dissemination outputs for young people, families, peer workers, and services.  - Co-planning with LE contributors to explore extending MyBRANCHES for earlier use in EIPS (e.g., at service entry), which has been identified through co-design. |

***Supplementary File 2. CONSORT 2025 Flow Diagram for Primary Effectiveness Outcome***

## Analysis

## Follow-up

Lost to follow-up for primary effectiveness outcome (12-week RAS-DS): (give reasons) (n=...)

Discontinued intervention (give reasons) (n=...)

## Enrolment

## Allocation

**MyPREP-ED intervention**

Allocated to MyPREP-ED intervention (Peer-coaching sessions plus MyBRANCHES tool (n=...)

♦ Received allocated intervention: (n=...)

♦ Did not receive allocated intervention (give reasons) (n=...)

Analysed (n=…)
♦ Excluded from analysis (give reasons) (n=...)

Excluded (n=...)

♦  Not meeting inclusion criteria (n=...)

♦  Declined to participate/did not consent (n=...)

♦  Judged at high risk (n=...)

♦  Other reasons (n=...)

Assessed for eligibility (n=...) (young people approaching EIPS discharge)

Enrolled and completed baseline (n=...)

Randomised (1:1) via REDCap, stratified by site + service stream^i^) (n=…)

Analysed (n=...)
♦ Excluded from analysis (give reasons) (n=...)

Lost to follow-up for primary effectiveness

outcome (12-week RAS-DS): (give reasons) (n=...)

Discontinued usual care (give reasons) (n=...)

**Usual care**

Allocated to usual care at EIPS discharge

(n=...)

♦ Received allocated usual care (n = …)

♦ Did not receive allocated usual care (give reasons) (n = …)

1. *Service Streams: First Episode Psychosis (FEP) or Ultra High Risk (UHR)*

***Supplementary File 3. MyPREP-ED Logic Model and Active Ingredients***


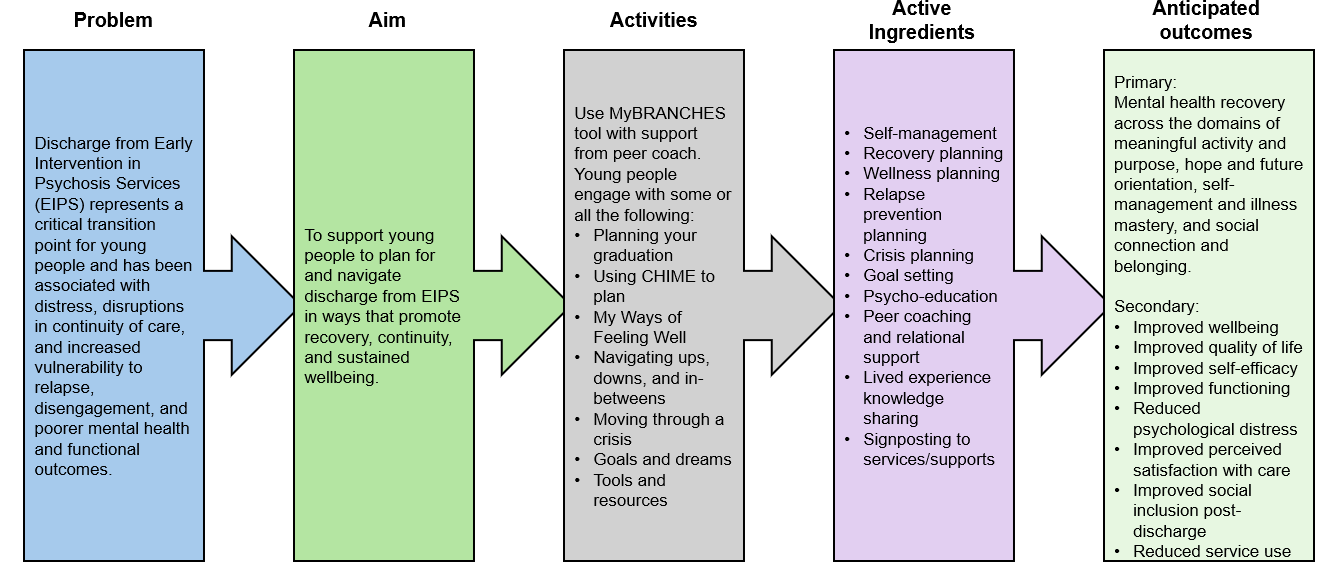


***Supplementary File 4. MyBRANCHES content overview***

***
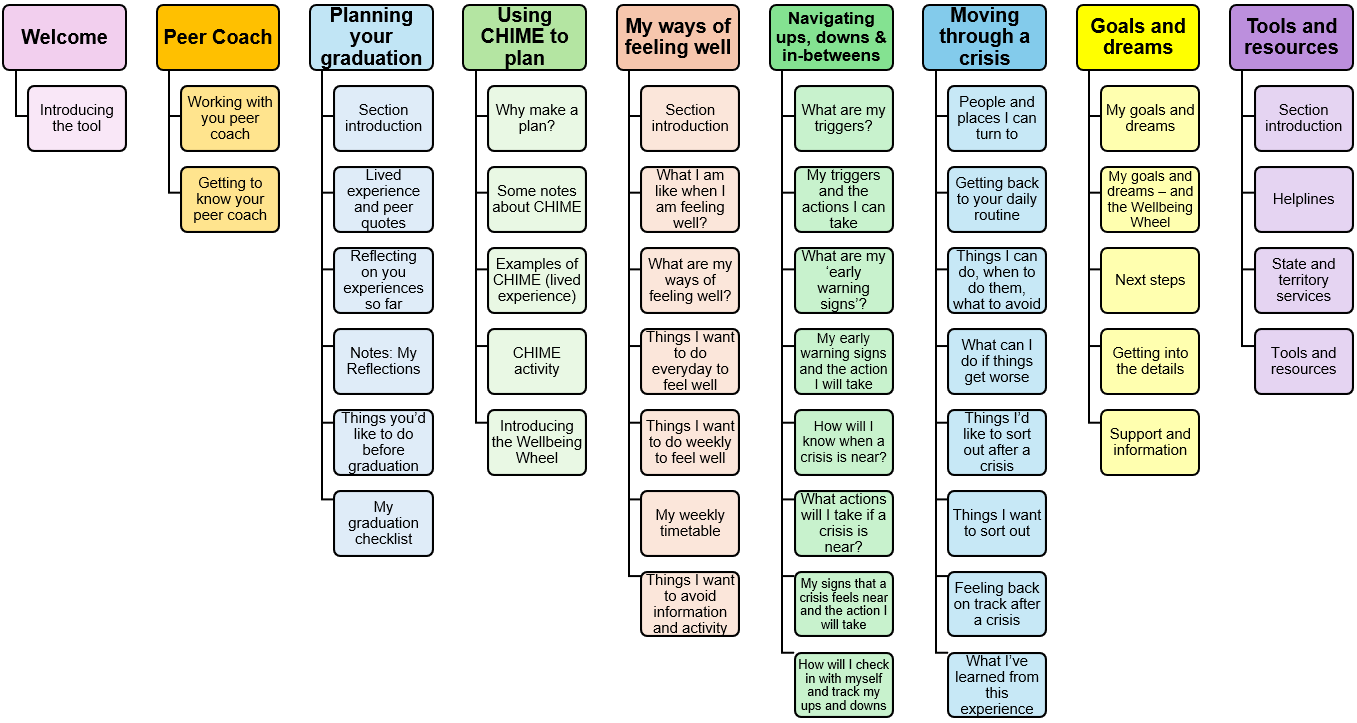
***

***Supplementary File 5. MyBRANCHES Interface Example***

***
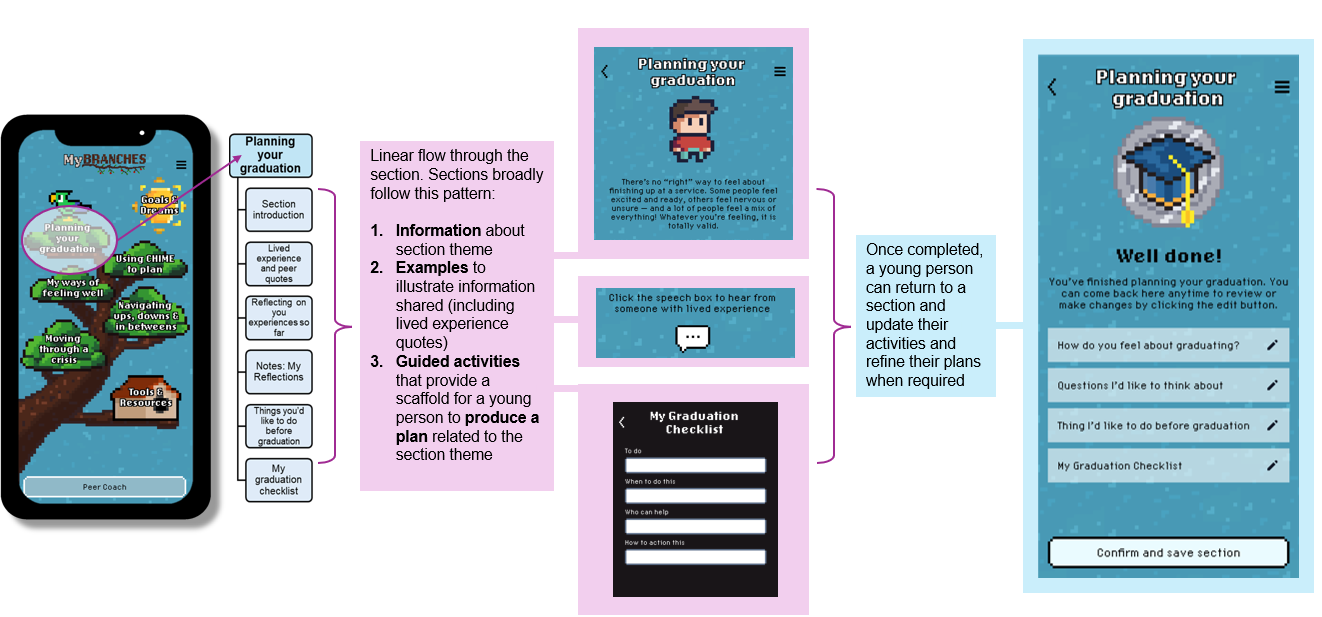
***
